# Proteome Analysis of Serum Purified Using *Solanum tuberosum* and *Lycopersicon esculentum* Lectins

**DOI:** 10.3390/ijms25021315

**Published:** 2024-01-21

**Authors:** Daisuke Nakajima, Ryo Konno, Yasuomi Miyashita, Masaki Ishikawa, Osamu Ohara, Yusuke Kawashima

**Affiliations:** 1 Department of Applied Genomics, Kazusa DNA Research Institute, 2-5-23 Kazusa Kamatari, Kisarazu 292-0818, Chiba, Japan; nakajima@kazusa.or.jp (D.N.); rkonno@kazusa.or.jp (R.K.); yasuomi.miyashita@chiba-u.jp (Y.M.); mishika@kazusa.or.jp (M.I.); ohara@kazusa.or.jp (O.O.); 2 Department of Developmental Biology, Graduate School of Medicine, Chiba University, 1-8-1 Inohana, Chuo-ku, Chiba 260-8670, Chiba, Japan

**Keywords:** proteome, *Solanum tuberosum* lectin, *Lycopersicon esculentum* lectin, serum, plasma, biomarker

## Abstract

Serum and plasma exhibit a broad dynamic range of protein concentrations, posing challenges for proteome analysis. Various technologies have been developed to reduce this complexity, including high-abundance depletion methods utilizing antibody columns, extracellular vesicle enrichment techniques, and trace protein enrichment using nanobead cocktails. Here, we employed lectins to address this, thereby extending the scope of biomarker discovery in serum or plasma using a novel approach. We enriched serum proteins using 37 different lectins and subjected them to LC–MS/MS analysis with data-independent acquisition. *Solanum tuberosum* lectin (STL) and *Lycopersicon esculentum* lectin (LEL) enabled the detection of more serum proteins than the other lectins. STL and LEL bind to N-acetylglucosamine oligomers, emphasizing the significance of capturing these oligomer-binding proteins when analyzing serum trace proteins. Combining STL and LEL proved more effective than using them separately, allowing us to identify over 3000 proteins from serum through single-shot proteome analysis. We applied the STL/LEL trace-protein enrichment method to the sera of systemic lupus erythematosus model mice. This revealed differences in >1300 proteins between the systemic lupus erythematosus model and control mouse sera, underscoring the utility of this method for biomarker discovery.

## 1. Introduction

Serum and plasma, collected via minimally invasive procedures, are the primary specimens used in clinical testing. In addition, serum and plasma are readily available in most biobanks, thus saving time and effort in specimen collection and facilitating biomarker discovery. Consequently, the discovery of new diagnostic markers from serum and plasma is actively pursued worldwide [1,2,3,4,5].

Despite extensive efforts in biomarker discovery via proteome analysis of serum and plasma, there has been limited success in identifying biomarkers suitable for clinical use. This limitation is attributed to the wide dynamic range of protein concentrations in serum and plasma, which hinders the analysis of low-abundance proteins necessary for comprehensive biomarker discovery [6,7]. The likelihood of discovering promising biomarkers will certainly increase with advancements in proteomics technology, enabling more comprehensive proteome analysis and elucidating a broader range of detectable proteins. Therefore, to identify biomarker proteins using mass spectrometry (MS), it is imperative to reduce this complexity and conduct extensive proteome analysis.

The depletion of highly abundant proteins, such as albumin, IgG, and transferrin, using antibody columns provides the most prevalent method for reducing the range of serum and plasma protein concentrations [8,9]. While effective, it is important to note that no antibody column can deplete all high-abundance proteins, making this method insufficient for detecting trace proteins in serum. To address this limitation, one approach for studying trace proteins in serum and plasma, avoiding interference from highly abundant proteins, involves collecting and analyzing extracellular vesicles via liquid chromatography-MS (LC–MS) [10,11,12]. In addition, the use of chemical nanoparticle cocktails for capturing trace serum and plasma proteins has recently gained attention [13,14,15]. Moreover, target-based analysis using antibodies and aptamers is also advancing, with services offered by Olink (Uppsala, Sweden) and SomaLogic (Boulder, CO, USA), enabling the simultaneous measurement of thousands of proteins in serum and plasma [16,17,18]. This indicates that various technologies are still under development to broaden the scope of biomarker discovery. 

To broaden the scope of biomarker discovery, we employed an alternative approach for enriching glycoproteins using lectins, with the aim of broadening the scope of biomarker discovery. More than 50% of the human proteome is estimated to be glycosylated [19]. Glycosylation is a highly complex post-translational modification that plays a pivotal role in various biological processes, such as cell differentiation, intercellular communication, and immunity [20,21]. In addition, many proteins identified as potential biomarkers for diseases, including cancer, are known to be glycosylated [22,23,24]. Therefore, comparative analysis of glycoproteins using LC–MS has the potential to reveal novel biomarkers that may not be detectable via conventional abundance-based comparisons. While the analysis of lectin-enriched proteins is promising, it is important to acknowledge that glycosylation occurs not only in trace proteins but also in high-abundance proteins in serum and plasma, which can complicate the detection of low-abundance proteins. However, we hypothesized that certain complex glycoforms might be less prevalent in highly abundant proteins and more common in trace proteins, such as in proteins leaking from tissues. We, therefore, used LC–MS to analyze serum proteins enriched using 37 different lectins, to determine which of the lectins enable the identification of a greater number of proteins. In addition, combinations of multiple lectins were examined, and a method for the enrichment of trace proteins using lectins was established. 

*Solanum tuberosum* lectin (STL) and *Lycopersicon esculentum* lectin (LEL) enabled the detection of more serum proteins than the other lectins. This innovative STL/LEL-based method achieved approximately 1.5-fold greater serum protein detection than the Top 14 Abundant Protein Depletion (TOP14D) method. Applying the STL/LEL enrichment method revealed that >1300 distinct proteins were enriched in systemic lupus erythematosus (SLE) model mice. The STL/LEL method was found to be effective for biomarker discovery.

## 2. Results and Discussion 

### 2.1. Enrichment of Trace Proteins in Serum Using Lectins

**Figure 1:**
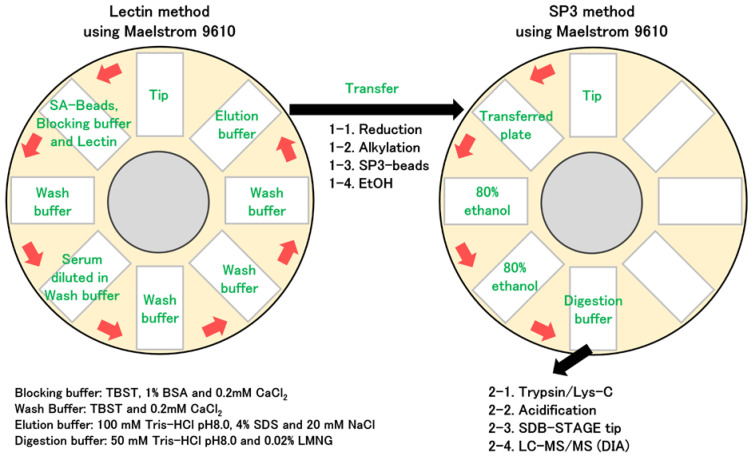
Automated method for the enrichment of trace serum proteins using biotinylated lectins. The Maelstrom 9610 instrument is equipped to accommodate eight different 96-deep-well plates, with one of these requiring specific tip placement for proper operation. Each plate can be maneuvered into position beneath a 96-pin magnetic head. This head descends into the 96-well plate, facilitating the binding, release, or mixing of magnetic beads in solution. Using the Maelstrom 9610 instrument, we automatically processed the reaction of biotinylated lectin with SA beads, the interaction of the lectin-bound beads with serum, and the requisite washing steps between each phase. Serum enriched using lectin was automatically treated via single-pot, solid-phase-enhanced sample preparation (SP3) using the Maelstrom 9610 instrument, following reduction and alkylation.

We first established a method for the automated enrichment of trace proteins from serum using lectins (Figure 1). Biotinylated lectins were coupled with streptavidin (SA) magnetic beads employing the Maelstrom 9610 instrument, an automated magnetic-bead manipulation device. This facilitated the concentration of lectin-binding proteins by enabling the interaction of the beads with serum. In addition, the SP3 method was automated using the Maelstrom 9610 instrument. This system allowed for the concurrent and automated processing of 96 samples, making it suitable for processing many samples such as clinical specimens. Owing to the limited variety of commercially available lectin-immobilized beads, we used biotinylated lectins, which are widely available on the market, to bind to the SA magnetic beads. Although our enrichment method primarily focuses on lectins, the same system could be applied to automate affinity purification using other biotinylated substances.

**Figure 2:**
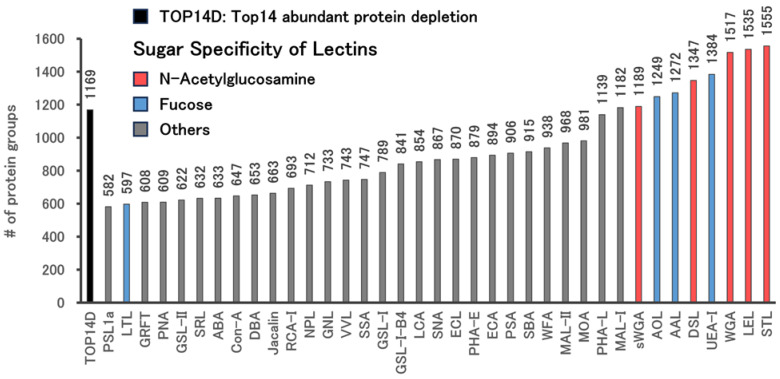
Number of proteins detected in sera enriched using 37 different lectins. As a representative of a typical method, serum subjected to Top 14 Abundant Protein Depletion (TOP14D) was also analyzed. The treated samples were analyzed via LC–MS/MS with a 60 min active gradient, using 200 ng of peptides, following trypsin digestion. The red, blue, and gray bars represent lectins with sugar specificity for N-acetylglucosamine, fucose, and others, respectively.

We utilized an automated method to capture trace proteins from serum using 37 different lectins (Table S1). For this experiment, commercially available pooled sera were employed. The proteins collected using the lectins were subsequently trypsin-digested and subjected to LC–MS/MS analysis (Figure 2 and Table S2). As a control, we analyzed serum treated with TOP14D resin. Among the lectins, STL, LEL, wheat germ agglutinin (WGA), *Ulex europaeus* agglutinin (UEA-I), *Datura stramonium* lectin (DSL), *Aleuria aurantia* Lectin (AAL), *Aspergillus oryzae* lectin (AOL), succinylated wheat germ agglutinin (sWGA), and *Maackia amurensis* Lectin I (MAL-I) identified more proteins than TOP14D. These lectins allowed for the enrichment of trace proteins beyond the typical performance of TOP14D. Moreover, these lectins exhibited the property of recognizing N-acetylglucosamine and fucose, except for MAL-I, which recognized other sugars. Of particular significance were STL and LEL, which identified many proteins and exhibited strong recognition of N-acetylglucosamine oligomers. Enrichment methods using STL, LEL, and WGA, which identified the most proteins, were validated, and the results were the same; i.e., STL was the top protein identifier, followed by LEL (Figure S1). Targeting these proved crucial for the identification of trace proteins in serum. 

**Figure 3:**
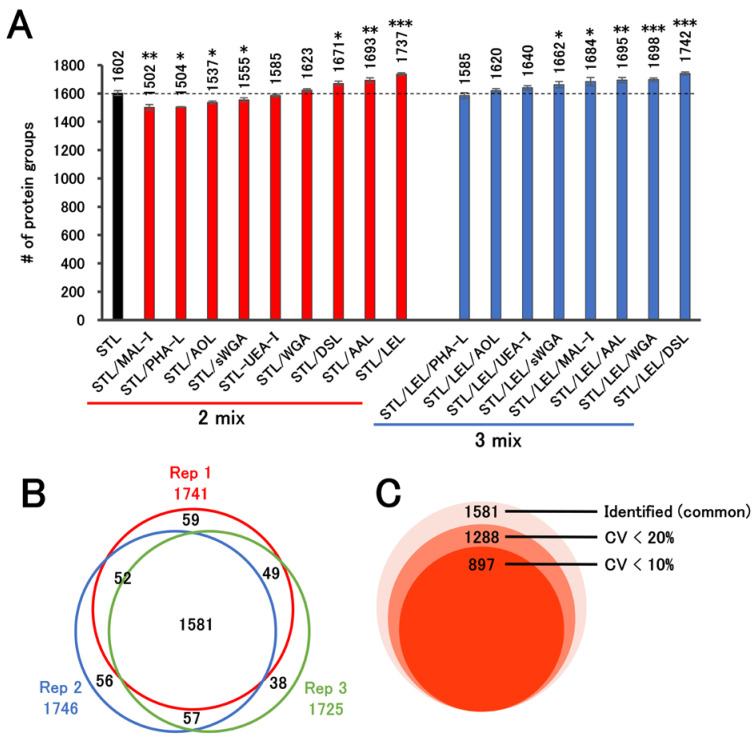
Combination of lectins used to detect trace proteins in serum. (**A**) Two-mix (red bar) and three-mix (blue bar) lectin combinations were tested. As a control, serum treated with *Solanum tuberosum* lectin (STL) alone was analyzed (black bar). The treated samples were analyzed via LC–S/MS with a 60 min active gradient using 200 ng of peptides, following trypsin digestion (each n = 3). Statistical analysis was performed using Welch’s t-test for comparison with STL only; *** *p* < 0.005, ** *p* < 0.01, * *p* < 0.05. (**B**) Overlap of proteins identified by the STL/LEL enrichment method (n = 3). (**C**) Evaluation of reproducibility based on the intensity of commonly identified proteins. The number of proteins below the defined CVs was calculated.

We then examined whether the number of proteins observed could be increased by combining STL, which identified the most proteins, with other lectins (LEL, WGA, UEA-I, DSL, AAL, AOL, sWGA, or MAL-I; Figure 3A). The combination of STL and LEL, the top performers in terms of protein identification, proved the most effective, surpassing the performance of STL alone. Although the increase in protein identification by the combined use of STL/LEL compared to STL alone was not large (approximately 8%), we consider such an improvement in protein identification, with little change in labor and cost, beneficial. In addition, we examined the effect of adding another lectin to STL and LEL. Among these combinations, STL/LEL/DSL identified the most proteins, but the increase was marginal relative to STL/LEL, and the difference between STL/LEL and STL/LEL/DSL was not statistically significant. Thus, there was no advantage to using three lectins in combination. On the basis of these results, we conclude that STL/LEL is the optimal choice for the trace-protein enrichment of serum using lectins. However, these investigations were conducted on average sera using one pooled serum and not on individual sera. Therefore, the possibility that the STL/LEL method may cause variations in protein identification in sera in each individual cannot be ruled out.

To confirm the reproducibility of the STL/LEL enrichment method, we investigated the overlap of proteins identified by this method in technical replicates, and 90% of the identified proteins overlapped (Figure 3B). The results showed that 90% of the identified proteins overlapped. In addition, quantitative reproducibility was evaluated for commonly identified proteins (Figure 3C). Protein intensities are typically normalized in quantitative comparisons. However, we did not perform normalization in this study to evaluate the variability of the enrichment method. More than 80% of the proteins commonly identified (in triplicate) had a CV of 20% or less, confirming the high reproducibility of the STL/LEL enrichment method.

**Figure 4:**
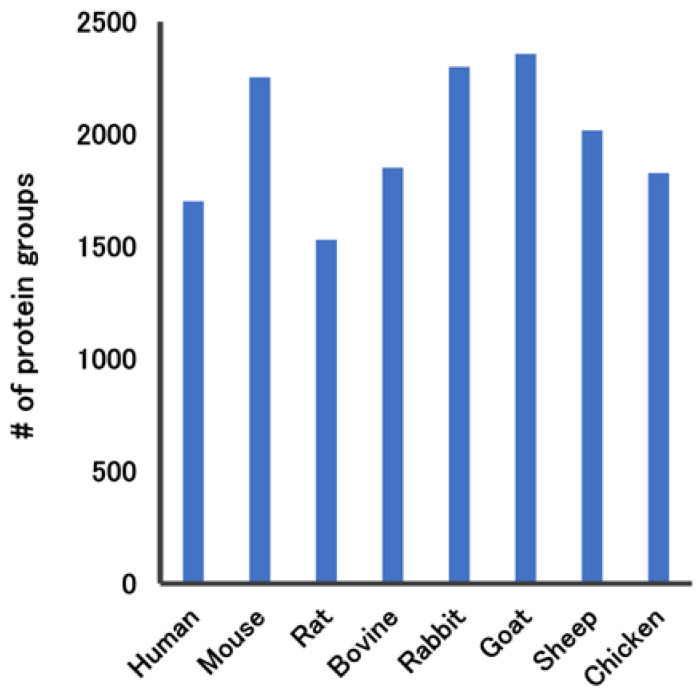
Number of proteins detected in *Solanum tuberosum* lectin (STL) and *Lycopersicon esculentum* lectin (LEL) (STL/LEL)-enriched sera from various species, including human, mouse, rat, bovine, rabbit, goat, sheep, and chicken sera. For each species, 200 ng of tryptic peptides was analyzed via LC–MS/MS with a 60 min active gradient.

We further investigated whether the STL/LEL enrichment method could be adapted to use samples from non-human animal species (Figure 4). This approach successfully identified more than 1500 proteins from various animal species, confirming that this method can enrich trace amounts of proteins, irrespective of the species. Since there are limited commercially available antibody-based depletion columns for species other than humans and mice, the ability to adapt this method to a wide range of species represents a significant advantage.

### 2.2. Deep Proteomics Using the STL/LEL Enrichment Method

**Figure 5:**
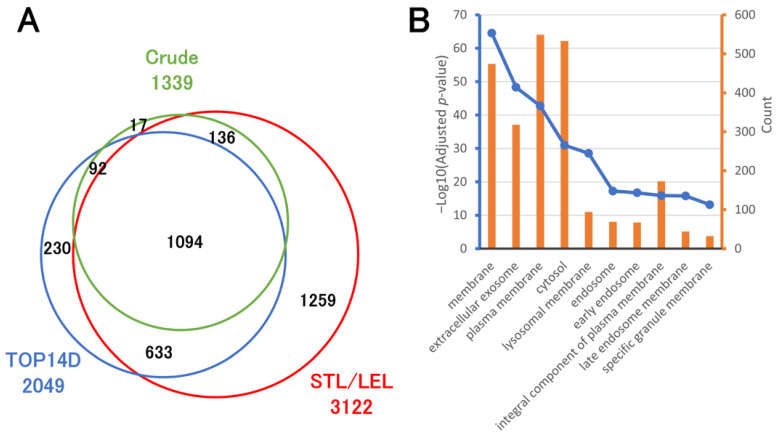
Comparison of proteins observed in crude, TOP14D-treated, and STL/LEL-enriched sera via 120 min active gradient LC-MS/MS. A total of 500 ng of tryptic peptides was analyzed. (**A**) Overlap of proteins identified by each treatment. (**B**) Cellular component of GO enrichment analysis for proteins observed only in STL/LEL-enriched serum.

We established the STL/LEL method, initially employing a 60 min active gradient, which was later extended to 120 min, with a focus on detecting trace serum proteins. Figure 5A and Table S3 compare the protein profiles of crude human serum, TOP14D-treated human serum, and STL/LEL-enriched human serum within this extended 120 min gradient. A total of 1339, 2049, and 3122 proteins were identified in the crude serum, TOP14D-treated serum, and STL/LEL-enriched serum, respectively. Gene Ontology (GO) analysis of proteins exclusively detected in the STL/LEL-enriched serum showed an enrichment of membrane proteins (Figure 5B). Protein identification results for proteins containing two or more unique peptides also confirmed that they were rich in membrane proteins (Figure S2). Since membrane proteins are known for their extensive glycosylation [25], we speculated that the STL/LEL method concentrated membrane proteins released into the serum. Furthermore, we confirmed that interleukin receptors, such as interleukin 1 receptor, type I (IL1R1), IL2RA, IL2RB, IL2RG, IL3RA, IL10RB, IL15RA, IL17RA, IL17RC, and IL27RA, were notably enriched through the STL/LEL method.

STL/LEL-enriched serum would ideally identify glycoproteins, but only 1446 of the 3122 proteins identified were found to be glycoproteins (Table S3). Although not all proteins have been fully investigated for glycosylation, glycoprotein addition was not confirmed in more than half of the STL/LEL-enriched sera identified. Since proteins are known to interact with other proteins, many of the non-glycosylated proteins may have contained proteins that interacted with glycoproteins enriched by STL and LEL. The primary objective of this study was to identify a large number of trace proteins in serum using lectins. The ability to detect many non-glycoproteins in addition to glycoproteins by excluding high abundances in serum using the STL/LEL method greatly improved protein identification.

**Figure 6:**
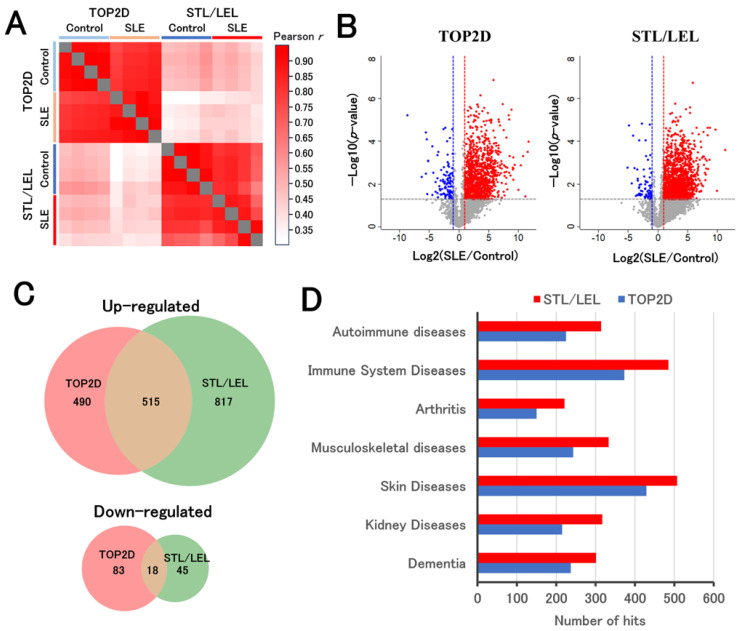
Analysis of proteins in the serum of SLE model and control mice using STL/LEL and TOP2D methods. (**A**) Pearson correlation coefficient heatmap for individual protein intensities between each sample. (**B**) Volcano plot of protein intensities obtained from sera of SLE model and control mice. The criteria for identifying altered proteins included a more than two-fold change with *p* < 0.05 (Welch test) between the two groups. The red dots indicate proteins upregulated in the sera of R-SLE model mice, and the blue dots indicate downregulated proteins in the sera of SLE model mice. (**C**) Overlap between altered proteins in SLE model mouse sera detected by STL/LEL and TOP2D methods. (**D**) Disease ontology enrichment analysis of proteins upregulated in the sera of the SLE model mice, with selected terms related to SLE and its complications.

We compared sera from four SLE model and four control mice using the STL/LEL and 120 min active gradient LC-MS/MS methods. To evaluate the STL/LEL method, we also treated the same samples with the top 2 abundant protein depletion (TOP2D) method. A comparison of protein profiles in each group showed that the differences between the STL/LEL and TOP2D methods were more significant than the differences between the groups of mice, confirming that the STL/LEL method differs from the conventional method (Figure 6A, Table S4). In addition, the mean Pearson *r* correlations for control sera treated with the TOP2 and STL/LEL methods were unchanged at 0.91 and 0.91, respectively, confirming that the STL/LEL method can stably treat sera from different individuals. The STL/LEL and TOP2D methods identified 1395 and 1106 proteins, respectively, that exhibited fluctuating concentrations in the sera of SLE model mice, with the STL/LEL method detecting a greater number of fluctuations (Figure 6B,C). Many of the altered proteins were unique to each method (Figure 6C), indicating that this method can reveal biomarkers from a different perspective than the TOP2D method. 

In addition, we conducted a disease enrichment analysis for proteins increased in SLE (Table S5). Figure 6D displays the results for disease terms associated with SLE and its complications. In all the terms shown, the STL/LEL method categorized more proteins and appeared to capture the essence of the disease more effectively compared to the TOP2D method. The STL/LEL method was able to observe the effects of disease-induced changes in glycoforms, which may enable it to discover biomarkers for diseases with many reported associations with glycosylation, such as cancer and inflammation. As SLE is an inflammation-related disease, the STL/LEL method may better distinguish disease features.

The reason for the large difference in fluctuating proteins between the STL/LEL method and TOP2D was that the STL/LEL method detected glycoproteins enriched by STL and LEL and proteins interacting with them, while it did not simply analyze protein abundance in the serum, in contrast to the TOP2D method. The fact that many differences in fluctuating proteins were detected indicates the uniqueness of the STL/LEL method. However, it is possible that the results of the STL/LEL method do not correlate with those of ELISA. Thus, in the absence of any correlation in a typical ELISA validation experiment, fast LC-MS/MS analysis using selected reaction monitoring of serum enriched by the STL/LEL method, or ELISA using a 96-well plate conjugated with STL/LEL and an antibody against the target protein, is required. Ninety-six-well plates coated with streptavidin are commercially available, and the biotinylated STL and LEL used in this study can be easily bound to the plates. We believe that it is possible to perform validation experiments on biomarker candidates found by the STL/LEL method for several hundreds of samples.

## 3. Materials and Methods

### 3.1. Serum

Three distinct lots of pooled human sera were purchased from KOHJIN BIO (Saitama, Japan). These three serum samples were combined and employed in the experiment. Normal sera from mice, rats, cattle, rabbits, goats, sheep, and chickens were purchased from TK Craft (Gunma, Japan). Additionally, sera from female (NZB × NZW) F1 hybrid mice with spontaneous polygenic autoimmune disease, serving as a systemic lupus erythematosus (SLE) model, and sera from pre-symptomatic mice (n = 4), serving as control mice (n = 4), were purchased from Hooke Laboratories (Lawrence, MA, USA).

### 3.2. Preparation of Serum Samples

In the lectin-enrichment method, glycoproteins were automatically enriched from serum using lectins and a Maelstrom 9610 instrument (Taiwan Advanced Nanotech, Taoyuan, Taiwan). Initially, 950 µL of blocking buffer (tris-buffered saline with Tween-20 (TBST), 1% bovine serum albumin (BSA), and 0.2 mM CaCl_2_ were combined with 50 µL of streptavidin (SA) bead suspension (CAT# 21152104010350; Cytiva, Marlborough, MA, USA). Subsequently, 40 µL of a 0.5 µg/µL lectin solution (Table S1) was added. For combinations of two lectins, 20 µL of each was used, and for three-lectin combinations, 13.3 µL of each was added. The mixture was agitated for 30 min, and the beads were then washed with wash buffer (TBST and 0.2 mM CaCl_2_). Next, 50 µL of serum, diluted in 150 µL of wash buffer, was added to the beads, followed by mixing for 60 min. The beads were subsequently washed three times with 1000 µL of wash buffer and mixed in 200 µL of 100 mM Tris-HCl (pH 8.0), 4% SDS, and 20 mM NaCl for 10 min.

For the depletion of high-abundance proteins, human serum was processed using Top14 Abundant Protein Depletion Mini Spin Columns (Thermo Fisher Scientific, Waltham, MA, USA), following the manufacturer’s instructions; mouse serum was treated with Proteome Purify 2 Mouse Serum Protein Immunodepletion Resin (R&D System, Minneapolis, MN, USA), as the manufacturer’s guidelines.

### 3.3. Protein Digestion

The treated serum was exposed to 20 mM tris(2-carboxyethyl) phosphine at 80 °C for 10 min, alkylated using 35 mM iodoacetamide at 25 °C for 30 min, and protected from light. Subsequently, it was subjected to cleaning and digestion with single-pot, solid-phase-enhanced sample preparation (SP3) [26,27] utilizing the Maelstrom 9610 instrument. Briefly, two types of Sera-Mag SpeedBead carboxylate-modified magnetic particles (hydrophilic particles, CAT# 45152105050250; hydrophobic particles, CAT# 65152105050250; Cytiva, Marlborough, MA, USA) were used. These beads were combined in a 1:1 (*v*/*v*) ratio, washed twice with distilled water, and reconstituted in distilled water to achieve a concentration of 10 μg solids/μL. Then, 20 μL of the reconstituted beads (SP3-beads) was added to the alkylated protein sample, followed by 99.5% ethyl alcohol to reach a final concentration of 75% (*v*/*v*), with mixing for 15 min. The supernatant was discarded, and the pellet was washed twice with 80% ethyl alcohol. The beads were subsequently resuspended in 80 μL of 50 mM Tris-HCl (pH 8.0) and 10 mM CaCl_2_ containing 0.02% lauryl maltose neopentyl glycol (LMNG) [28]. Thereafter, 500 ng of trypsin/Lys-C Mix (CAT# V5072, Promega, Madison, WI, USA) was gently mixed with the sample at 37 °C overnight for protein digestion. The digested sample was acidified with 20 μL of 5% trifluoroacetic acid (TFA) and sonicated using a Bioruptor II (CosmoBio, Tokyo, Japan) at a high level for 5 min at room temperature. The sample was desalted using a styrene divinylbenzene polymer (SDB) stop-and-go extraction (STAGE) tip (SDB-STAGE tip; GL Sciences, Tokyo, Japan), which was washed with 25 μL of 80% acetonitrile (ACN) in 0.1% TFA, followed by equilibration with 50 μL of 3% ACN in 0.1% TFA. The sample was loaded onto the tip, washed with 80 μL of 3% ACN in 0.1% TFA, and eluted with 50 μL of 36% ACN in 0.1% TFA. The eluate was dried in a centrifugal evaporator (miVac Duo concentrator; Genevac, Ipswich, UK). The dried sample was then redissolved in 0.01% decyl maltose neopentyl glycol (DMNG) containing 0.1% TFA. The redissolved sample was assayed for peptide concentration using a Lunatic instrument (Unchained Labs, Pleasanton, CA, USA) and transferred to an LC vial (Thermo Fisher Scientific, Waltham, MA, USA).

### 3.4. LC–MS/MS with Data-Independent Acquisition (DIA)

The redissolved peptides were injected directly onto a 75 μm × 12 cm nanoLC column (Nikkyo Technos Co., Ltd., Tokyo, Japan) at 50 °C and separated using a 60 min gradient (A = 0.1% formic acid (FA) in water, B = 0.1% FA in 80% can) with the following settings: 0 min at 6% B, 50 min at 36% B, 57 min at 70% B, and 60 min at 70% B, all at a flow rate of 200 nL/min. This separation was performed using an UltiMate 3000 RSLCnano LC system (Thermo Fisher Scientific). The eluting peptides from the column were then analyzed on a Q-Exactive HF-X (Thermo Fisher Scientific) with InSpIon system [29]. For DIA, the precursor range was defined based on previously established parameters [30]. MS1 spectra were collected in the range of 495–745 *m*/*z* at a resolution of 15,000, with an automatic gain control target of 3 × 10^6^ and a maximum injection time of 23 ms. MS2 spectra were collected at more than 200 *m*/*z* at a resolution of 30,000, with an automatic gain control target of 3 × 10^6^, a maximum injection time set to “auto”, and a normalized collision energy of 23%. The isolation width for MS2 was set to 4 *m*/*z*. For the 500–740 *m*/*z* window pattern, an optimized window arrangement was employed using Scaffold DIA v. 3.2.0Proteome Software, Inc., Portland, OR, USA).

For comprehensive proteome analysis, redissolved peptides were injected directly onto a 75 μm × 30 cm nanoLC column (CoAnn Technologies, Richland, WA, USA) at 60 °C. The separation was conducted using a 120 min gradient (A = 0.1% FA in water, B = 0.1% FA in 80% ACN) with the following settings: 0 min at 3% B, 108 min at 33% B, 114 min at 65% B, and 120 min at 65% B, all at a flow rate of 200 nL/min, employing an UltiMate 3000 RSLCnano LC system. The peptides eluted via the column were subsequently analyzed on an Orbitrap Exploris 480 equipped with an InSpIon system. MS1 spectra were collected in the range of 495–745 *m*/*z* at a resolution of 15,000, with an automatic gain control target of 3 × 10^6^ and a maximum injection time set to “auto.” For MS2 spectra, a range of 200–1800 *m*/*z* was employed at a resolution of 45,000, with an automatic gain control target of 3 × 10^6^, a maximum injection time of “auto”, and stepped normalized collision energies set at 22, 26, and 30%. The isolation width for MS2 was configured at 4 *m*/*z*, and for the 500–740 *m*/*z* window pattern, an optimized window arrangement was utilized in Scaffold DIA.

### 3.5. Data Analysis

The DIA-MS data were queried against the in silico human or mouse spectral library, using DIA-NN v. 1.8.1 [31]. Initially, the spectral library was generated from the UniProt protein sequence database for either humans or mice using DIA-NN. The parameters for spectral library generation included trypsin as the digestion enzyme, allowance for 1 missed cleavage, a peptide length range of 7–35, precursor charge ranging from 2 to 4, precursor *m*/*z* ranging from 495 to 745, and fragment ion *m*/*z* ranging from 200 to 1800. Additionally, “FASTA digest for library-free search/library generation”, “deep learning-based spectra, RTs, and IM prediction”, “n-term M excision”, and “C carbamidomethylation” were enabled. For the DIA-NN search, the following parameters were applied: a mass accuracy of 10 ppm, MS1 accuracy of 10 ppm, protein inference based on genes, utilization of neural network classifiers in single-pass mode, quantification strategy using robust LC (high precision), cross-run normalization set to “off”. Furthermore, “unrelated runs”, “use isotopologues”, “heuristic protein inference”, and “no shared spectra” were enabled, while the use of match-between-run was deactivated. The threshold for protein identification was set at 1% or less for both precursor and protein false-discovery rate. Protein quantification values were aggregated over the quantification values of unique peptides as calculated by DIA-NN. Log_2_ transformation of protein intensity was applied, and a filtering step was conducted to ensure that for each protein, at least one group contained a minimum of 70% valid values. Missing values were imputed using random numbers drawn from a normal distribution (width, 0.3; downshift, 1.8) using Perseus v1.6.15.0 [32]. The criteria for identifying altered proteins included a more than two-fold change with *p* < 0.05 (Welch test) between the two groups. GO enrichment analysis was conducted using DAVID (https://david.ncifcrf.gov/tools.jsp (3 December 2023)), and disease ontology enrichment analysis (based on the HumanPSD database) was conducted using the geneXplain platform (GeneXplain GmbH, Wolfenbüttel, Germany).

## 4. Conclusions

To broaden the scope of serum biomarker discovery, we established an automated method that combines STL and LEL for enriching trace proteins in serum. The STL/LEL-based method demonstrated an approximately 1.5-fold increase in the detection of serum proteins compared to the TOP14D method. Furthermore, comparing sera from SLE mice and control mice via the STL/LEL-based method revealed over 1300 distinct proteins. By identifying proteins with N-acetylglucosamine oligomers using STL and LEL, we observed variations that generally differ from the depletion method, indicating the effectiveness of our proof-of-concept approach for biomarker discovery.

## Data Availability

The mass spectrometry proteomics data were deposited in the ProteomeXchange Consortium via the jPOST partner repository with the dataset identifiers PXD047848 and PXD048327 for ProteomeXchange and JPST002420 and JPST002449 for the jPOST.

## Supplementary Materials

The supporting information can be downloaded at: https://www.mdpi.com/article/10.3390/ijms25021315/s1.
