# Supplementary figures and images for: Proteome Analysis of Serum Purified Using Solanum tuberosum and Lycopersicon esculentum Lectins
Source: Int J Mol Sci. 2024 Jan 21;25(2):1315. doi: 10.3390/ijms25021315 (PMC10816257; doi:10.3390/ijms25021315)

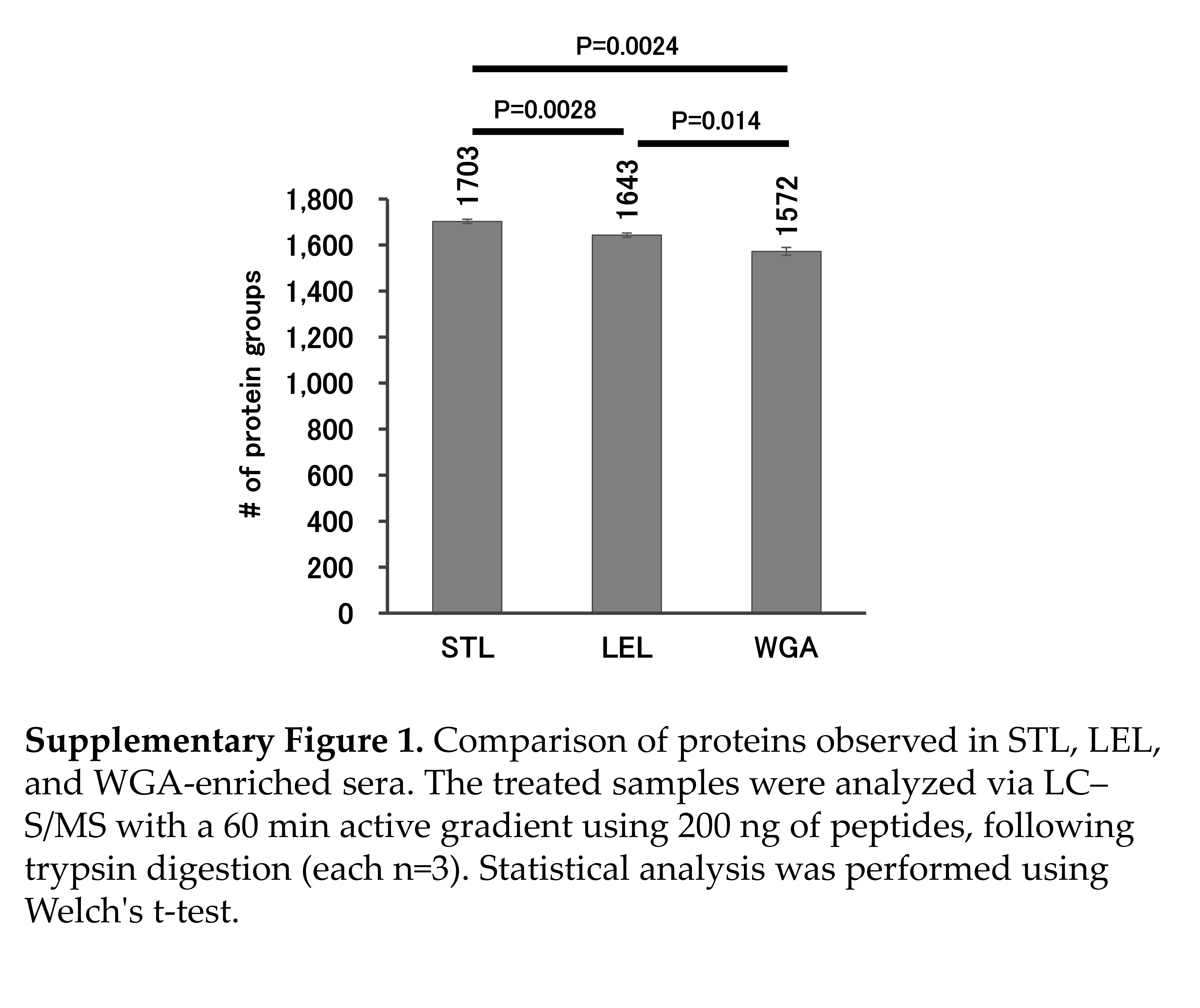

Supplement: Supplementary file 1 [file ijms-25-01315-s001.zip › Supplementary Figure 1.TIF]

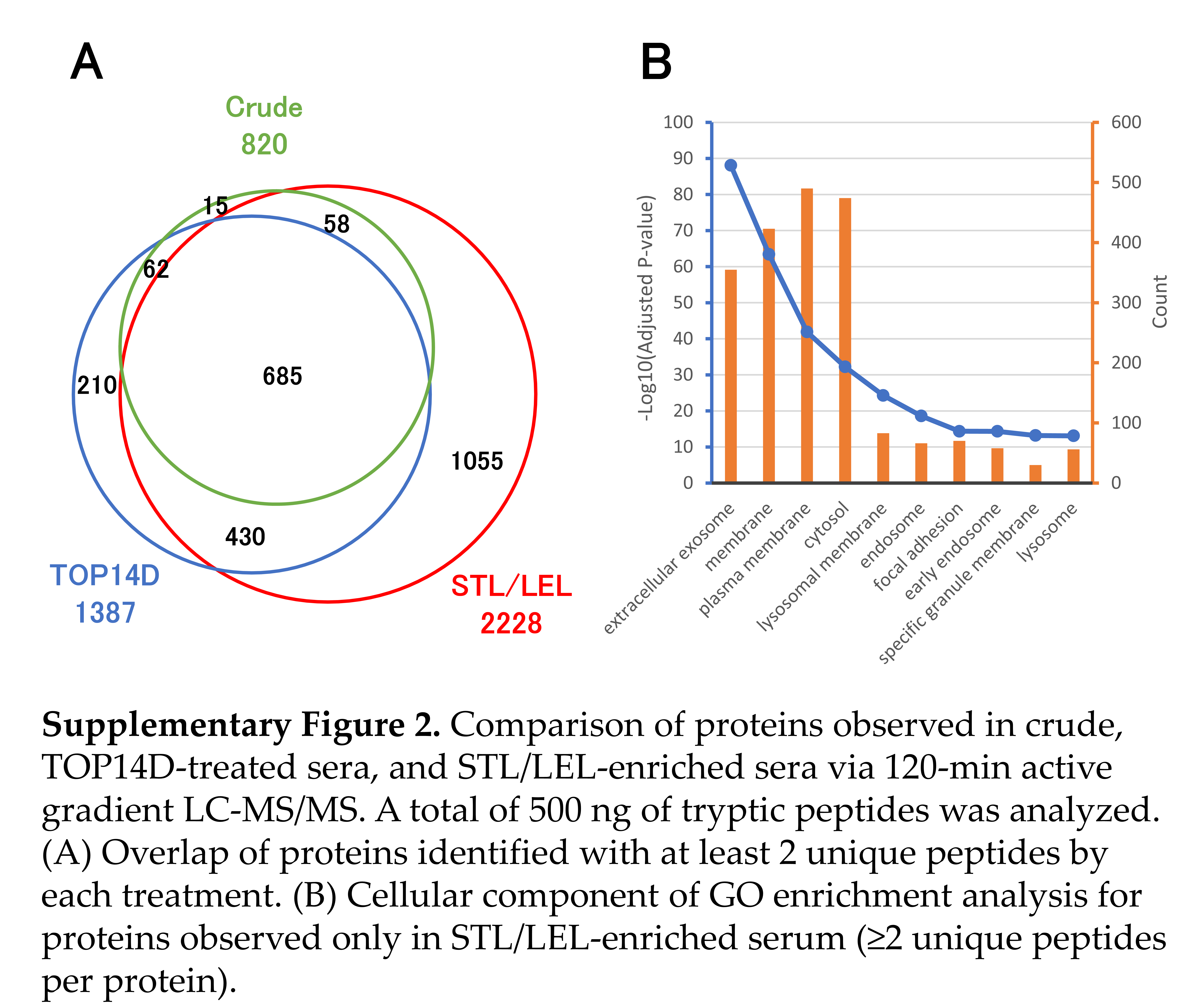

Supplement: Supplementary file 1 [file ijms-25-01315-s001.zip › Supplementary Figure 2.TIF]
